# In vivo and in situ evaluation of innovative approaches in dentin hypersensitivity treatment

**DOI:** 10.1186/s12903-025-05865-y

**Published:** 2025-04-18

**Authors:** Heba Abd El-Fattah Mohamed, Dina Ezzeldin Mohamed, Elhassan Hassanein, Heba El-din Salah El-din Hamza

**Affiliations:** 1 Conservative Dentistry Department, Faculty of Dentistry, Cairo University, 11 El- Saraya St, Manial, Cairo 11553 Egypt; 2 Heinrich Heine Universität Düsseldorf, Düsseldorf, Germany; 3 Conservative Dentistry Department, School of Dentistry, New Giza University, 6th of October City, Giza Egypt

**Keywords:** Dentin hypersensitivity, PRG barrier coat, Giomer, Embrace, Xylitol-coated calcium phosphate, Sodium fluoride, Duraphat, Dentinal tubules occlusion, Dentinal tubules obliteration

## Abstract

**Background:**

Dentin hypersensitivity (DH) causes transient sharp pain from exposed dentinal tubules, adversely affecting oral health and quality of life. This study compared the efficacy of two innovative treatments against Sodium Fluoride Varnish in reducing DH and occluding dentinal tubules over eight weeks.

**Methods:**

This randomized, triple-blind, three-parallel-arm clinical and in situ study included a total of 63 participants (age range: 26–46 years), each randomly assigned to one of three treatment groups: PRG Barrier Coat, Embrace varnish, or Duraphat varnish. The clinical trial assessed pain intensity was assessed using Visual Analog Scale (VAS) after tactile, evaporative, and thermal stimuli at baseline, 3 min, 2 weeks, 4 weeks, and 8 weeks. The in-situ phase evaluated dentinal tubules occlusion pre- and post-treatment using scanning electron microscopy (SEM) at 2000× magnification. Statistical Analysis was conducted using Kruskal-Wallis and Friedman tests for intergroup and intragroup comparisons, respectively, and Spearman’s correlation for pain reduction-tubule occlusion relationship (*p* < 0.05).

**Results:**

PRG Barrier Coat achieved the highest efficacy with 94.9% pain reduction and 96.9% tubule occlusion. Embrace varnish showed moderate results with 64.3% pain reduction and 69.7% tubule occlusion, while Duraphat varnish provided limited performance with 45.4% pain reduction and 48.3% tubule occlusion. PRG Barrier Coat exhibited the most prolonged effects in reducing dentin hypersensitivity, aligning with its higher tubule occlusion. Embrace varnish demonstrated moderate performance, showing initial pain relief that was less sustained over time. Duraphat varnish provided the least reduction in pain and tubule occlusion, with effects that appeared transient.

**Conclusions:**

This study demonstrated that PRG Barrier Coat and Embrace varnish effectively reduced pain intensity and promoted dentinal tubule occlusion, with PRG Barrier Coat showing the most sustained effects. These findings highlight the importance of dentinal tubule occlusion in DH management and suggest that treatment selection should consider both immediate pain relief and durability of therapeutic effects.

**Trial registration:**

ClinicalTrials.gov (NCT04568473) on September 23, 2020.

## Background

Dentin hypersensitivity (DH) is a prevalent clinical condition characterized by transient, sharp pain arising from exposed dentin in response to thermal, evaporative, tactile, osmotic, or chemical stimuli which resolves upon stimulus removal [1]. This condition significantly impacts daily activities and oral health-related quality of life [2]. While current DH therapies effectively reduce pain and improve psychological well-being, they often provide only temporary relief, highlighting the need for durable and effective treatment strategies [3].

The hydrodynamic theory explains DH as fluid movement within exposed dentinal tubules stimulating nerve endings and generating pain [4, 5]. Smear layer removal further increases dentinal permeability, exacerbating hypersensitivity [6]. Therefore, DH treatment modalities primarily focus on either modulating nerve excitability or occluding dentinal tubules to reduce fluid movement and associated pain [7].

Fluoride varnishes are a widely accepted treatment for DH management, primarily acting through calcium fluoride precipitation, which occludes dentinal tubules and reduces dentin hypersensitivity. While fluoride varnishes provide effective short-term relief, their effects are often transient, requiring frequent reapplications to maintain efficacy [8].

Novel bioactive materials offer potential advancements in DH treatment by combining tubule occlusion with bioactive ion release. PRG Barrier Coat is a resin-based varnish incorporating surface pre-reacted glass-ionomer (S-PRG) filler technology. It provides a durable polymeric seal while releasing multiple bioactive ions that contribute to DH relief [5, 6, 8]. Embrace varnish combines sodium fluoride with CXP technology, utilizing xylitol-coated calcium and phosphate ions to enhance fluoride release and promote remineralization [9].

Despite their distinct mechanisms of action, both fluoride-based and bioactive materials are clinically used for DH management, making their comparative evaluation essential. This study addresses a gap in current research by directly comparing these approaches to determine their effectiveness in pain relief and dentinal tubule occlusion.

While these innovative materials show promise in DH management, their clinical effectiveness in pain relief and dentinal tubule occlusion over extended periods remains an area of ongoing investigation. Further research is needed to optimize their clinical application and establish evidence-based treatment protocols.

This study aimed to compare the effectiveness of PRG Barrier Coat and Embrace varnish against Duraphat varnish in managing DH, with a focus on pain relief and dentinal tubule occlusion. The primary objective was to assess pain intensity at baseline, 3 min, 2 weeks, 4 weeks, and 8 weeks using the Visual Analog Scale (VAS). The secondary objective was to evaluate dentinal tubule occlusion using scanning electron microscopy (SEM) by analyzing pre- and post-treatment dentin surfaces. Additionally, the study aimed to compare the performance of these three materials in reducing pain sensitivity and occluding dentinal tubules over time, determining whether there were statistically significant differences among them.

The null hypothesis proposed that there would be no statistically significant differences in pain intensity (measured by VAS for tactile, evaporative, and thermal stimuli) among PRG Barrier Coat, Embrace varnish, and Duraphat varnish at 3 min, 2-week, 4-week, and 8-week intervals.

## Methods

### Trial design

**Fig. 1:**
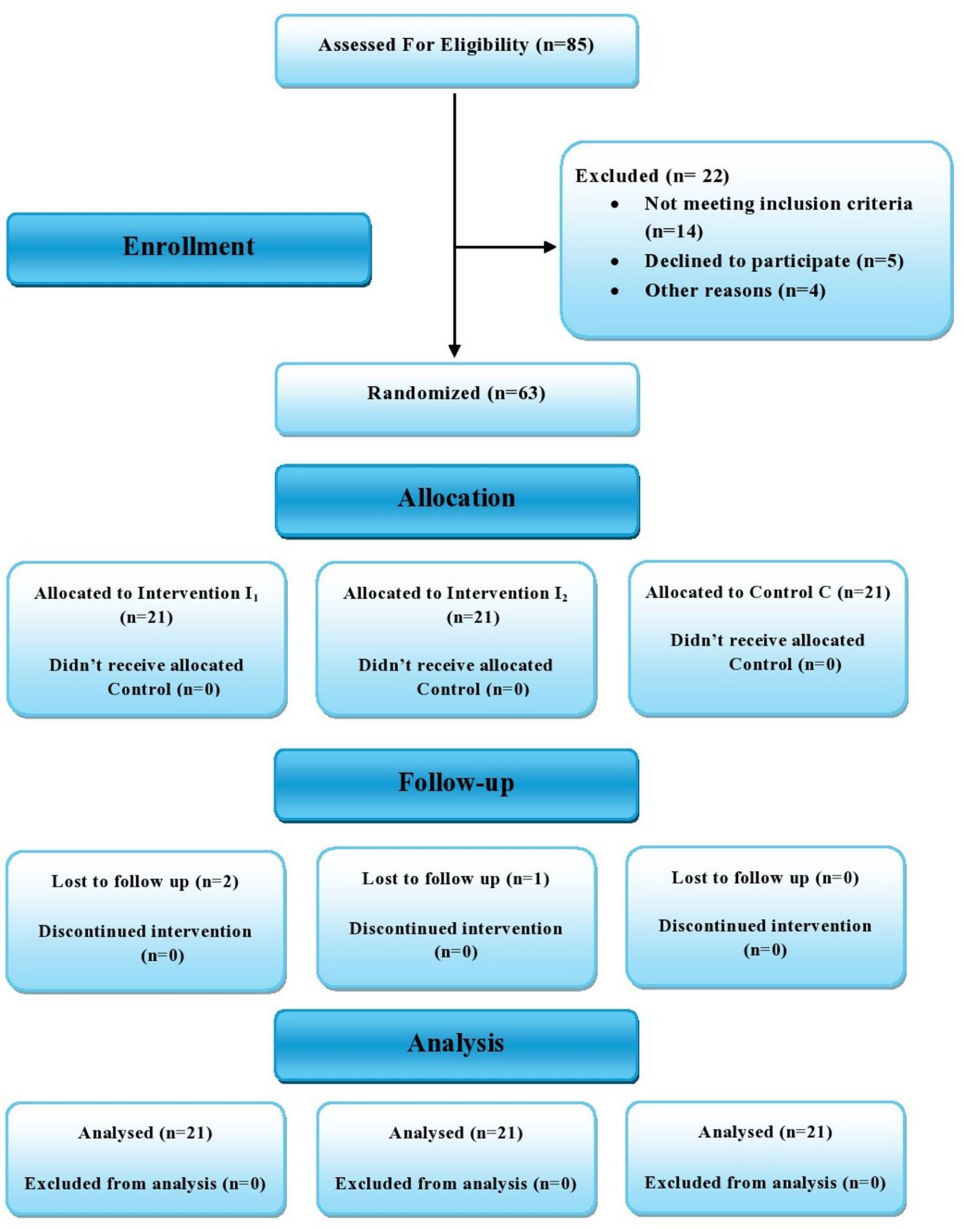
CONSORT flow diagram

This was a randomized, triple-blind, three-parallel-arm clinical trial at Cairo University’s Faculty of Dentistry. The study design adhered to the guidelines specified in the Consolidated Standards of Reporting Trials (CONSORT) statement (Fig. 1).

### Trial registration and ethical approval

This research was ethically approved by Cairo University’s Faculty of Dentistry Research Ethics Committee (REC #19-9-20) in accordance with the latest version of the Declaration of Helsinki. The trial was registered with ClinicalTrials.gov (NCT04568473) on September 23, 2020.

### Sample size calculation

The sample size was determined based on a previous study by Suri et al. (2016) [10], which reported the mean and standard deviation values of pain intensity following tactile stimulation after two months in the sodium fluoride varnish group were found to be 1.23 (SD = 1.006). A Cohen’s d effect size of 1.0 was estimated to detect a minimum clinically important difference of 1 pain score between the study groups, assuming a Type I error probability (α) of 0.05 and a study power of 0.8 (80%). The calculated sample size was 17 subjects per group, which was increased by 20% (to 21 subjects per group) to compensate for possible dropouts. The sample size calculation was performed using G Power software (version 3.1.9.7).

### Eligibility criteria

All participants were selected and clinically examined in accordance with the predefined inclusion and exclusion criteria presented in Table 1.

**Table 1:** Inclusion and exclusion criteria of participants and teeth

Inclusion criteria of participants	Exclusion criteria of participants
• Adult participants aged from 20 to 50 years of age. • Participants had good oral hygiene. • Participants had cervical dentin hypersensitivity. • Participants had clinically sound first upper molars. • Participants were willing to cooperate and attend recall appointments.	• Participants had poor oral hygiene. • Pregnant or lactating women. • Concurrent participation in other research studies. • Inability to comply with study procedures. • History of allergic reactions to study materials. • Medical conditions interfering with pain reporting accuracy (pain disorders, constant use of analgesics, anti-inflammatory, or psychotropic medications). • Use of desensitizing products within the past three months. • Undergoing orthodontic treatment. • History of periodontal surgeries within the last six months. • Periodontitis and pulpitis. • Carious, crowned, or missing upper first molars.
**Inclusion criteria of teeth**	**Exclusion criteria of teeth**
• Hypersensitive cervical areas on facial surfaces of incisors, cuspids, and bicuspids, with exposed cervical dentin and (VAS) pain score ≥ 5. • Teeth with Non-Carious Cervical Lesions.	• VAS pain score < 5. • Teeth displaying significant untreated dental conditions (periodontitis, rampant caries). • Teeth with defects causing pain unrelated to hypersensitivity.

### Recruitment and informed consent

Each participant’s detailed personal, medical, and dental histories were documented before clinical examination. All participants received comprehensive explanations about study details, including objectives, procedures, potential risks/benefits, safety measures and expected duration of participation. Eligible subjects were then required to sign an Arabic informed consent form, approved by Cairo University’s Faculty of Dentistry REC, detailing the trial’s ethical considerations and ensuring voluntary participation.

### Group allocation

Participants were randomized into three groups - PRG barrier coat (Group I1), Embrace Varnish (Group I2), and Duraphat Varnish (Group C).

### Random sequence generation

Simple randomization was conducted using a random sequence generator provided by Randomness and Integrity Services Ltd. (https://www.random.org/), assigning numbers 1–21 to Group I1, 22–42 to Group I2, and 43–63 to Group C.

### Allocation concealment

Opaque sealed envelopes containing group assignments were prepared by an independent individual who was not involved in the trial. The principal investigator recorded allocations electronically while all allocation records were securely stored to maintain confidentiality.

### Blinding

A triple-blind protocol was implemented wherein participants, assessors, and statistician were unaware of group assignments. The operator couldn’t be blinded due to distinct presentation and application techniques of the materials. Participants remained unaware of their group allocation throughout the trial, preventing their knowledge from influencing their perception of hypersensitivity, thus maintaining the integrity of the study’s outcomes.

The total number of participants assessed for eligibility, recruitment, randomization, allocation, and evaluation is detailed in the CONSORT flow diagram (Fig. 1).

### Materials

The materials used in this trial were BioSmart Light Cured Protective Shield with bioactive S-PRG filler technology (PRG Barrier Coat, SHOFU, Kyoto, Japan), 5% Sodium Fluoride with Xylitol-coated Calcium and Phosphate (CXP) Varnish (Embrace Varnish, Pulpdent, Watertown, MA, USA) and 5% Sodium Fluoride Varnish (Duraphat, Colgate Palmolive, New York, USA). A full description of each material is presented in Table 2.

**Table 2:** Materials used in the study

Material	Specification	Composition	Manufacturer	LOT#
PRG Barrier Coat	BioSmart Light Cured Protective Shield with bioactive S-PRG filler technology	Base: S-PRG filler based on fluoroboroaluminosilicate glass, Distilled water, Methacrylic acid monomer, and others. Active: Phosphonic acid monomer, Methacrylic acid monomer, Bis-MPEPP, Carboxylic acid monomer, TEGDMA, Polymerization initiator, and others.	SHOFU, Kyoto, Japan	102,001
Embrace™ Varnish	5% Sodium Fluoride with Xylitol-coated Calcium and Phosphate (CXP) Varnish	Hydrogenated rosin (< 35%), Ethanol (< 20%), Sodium fluoride (5%), amorphous fumed silica (< 3%), Xylitol-coated Calcium and Phosphate.	Pulpdent, Watertown, MA, USA	201,214
Duraphat^®^ Varnish	5% Sodium Fluoride Varnish	1 mL suspension contains 50 mg sodium fluoride (5% w/v), equivalent to 22,600 ppm fluoride ion (22.6 mg of fluoride) in an alcoholic solution of natural resins. Colophonium ( > = 30 -< 40), Ethanol (Ethyl Alcohol) ( > = 20 -< 30), Sodium Fluoride ( > = 3 -< 5), White beeswax (E901), Shellac (E904), Mastic, Saccharin(E954), Raspberry Flavour (which contains Ethyl Butyrate, Geraniol, Iris Resinoid, Isoamyl Acetate, Jasmine Absolute, Vanillin and Propylene Glycol).	Colgate Palmolive, New York, USA	10960BKNS6

Bis-MPEPP: 2,2-bis (4-methacryloxy polyethoxyphenyl) propane

TEGDMA: Triethylene glycol dimethacrylate

### Clinical procedures

#### Patient Preparation (wash-out period and preoperative instructions)

Following clinical examination and provision of informed consent, all participants underwent a two-week wash-out period before testing. During this period, participants were allowed to discontinue use of any desensitizing products and adhered to a standardized home-care regimen provided by researcher to ensure consistency throughout the study [1, 11, 12]. The standardized regimen included soft manual toothbrush, fluoride-free toothpaste (Miswak herbal toothpaste, Dabur India Limited, Uttar Pradesh, India) and a dental floss (oral-B essential floss (Waxed floss), Procter and Gamble, Cincinnati, OH 45202, USA) were used.

Participants were provided with comprehensive instructions to ensure adherence to study protocols. These instructions were delivered both verbally and in writing, covering standardized oral hygiene instructions and dietary counseling [6, 13, 14]. Oral hygiene guidance emphasized the correct brushing technique (Bass technique), the appropriate use of assigned toothpaste, toothbrushes, and dental floss, and adherence to a twice-daily brushing routine while dietary standardization guidelines, including restrictions on acidic foods and beverages [19]. The objective was to reduce or eliminate factors that could contribute to the progression of cervical dentin hypersensitivity and to standardize oral hygiene measures during the study period. Consistent with established guidelines, participants received standardized instructions before randomization, avoiding individualized oral health counseling during the study period to prevent bias [1]. Additionally, prophylactic oral care, including scaling and polishing procedures, was performed to ensure baseline oral health [14]. All dental concerns that could transiently influence dentin hypersensitivity such as mild gingivitis, defective restorations, or early-stage caries were identified and managed before trial initiation to ensure stable oral conditions and minimize external influences on the outcomes [14]. Without proper adherence to these preventive measures, external factors such as oral hygiene habits, dietary choices, and pre-existing conditions may act as confounders, potentially influencing treatment efficacy and outcomes.

#### Dentin hypersensitivity assessment

Dentin hypersensitivity (DH) was assessed using a combination of stimuli and a 10 cm horizontal VAS which was anchored by two verbal descriptors: “no pain” to “worst imaginable pain” [11]. Modified VAS, incorporating both numerical and visual elements, was employed to further enhance participant comprehension and ease of use. Pain intensity was categorized into four levels: (0 = no pain), 1– 3 = mild pain, 4–6 = moderate pain, and 7–10 = severe pain). The scale also included color-coded facial expression illustrations corresponding to each pain intensity level allowing participants to select the facial expression that best reflected their current pain experience [15, 16]. To ensure consistency and accuracy in the assessment process, participants were provided with VAS plastic cards that minimized verbal or emotional influence from the assessor, standardizing the evaluation method throughout the study [15].

Tactile test was conducted using a sharp dental explorer which was gently applied to exposed l dentin surface in a mesio-distal direction employing short strokes perpendicular to tooth’s long axis [14, 17, 18].

Evaporative test involved applying a continuous air blast using a conventional dental air–water syringe. Standardization of test parameters included maintaining the air pressure of 50 psi, the air temperature at approximately 20 °C and the syringe nozzle position 3 mm away from exposed dentin surface. Adjacent teeth were isolated using cotton rolls to prevent interference with measurement of the target tooth [1, 4, 19, 20].

For the thermal sensitivity test, a refrigerant spray (Endo Frost, Roeko, Coltene/Whaledent, Germany) was applied to a standardized 3 mm-diameter cotton pellet (size 4) (Richmond’s Cotton Pellets, Richmond Dental & Medical, USA*)*. The pellet, secured in a carrier, was gently placed on the exposed dentin surface for ≤ 5 s [1].

Each test was applied 1–5 s depending on the participant’s pain response, with mandatory 5-minute recovery intervals between tests to allow sufficient time for the tooth to recover ensuring consistency across evaluations [8, 21]. Participants marked VAS after each stimulus.

#### Assessor calibration

Two blinded expert assessors (R.H., E.M.) conducted DH evaluations using standardized criteria to ensure consistency and reliability. Calibration sessions were held on ten patients, who were not part of the trial, were evaluated to refine the assessment protocol and resolve any discrepancies through consensus discussions [14].

Inter- and intra-assessor reliability achieved 97% and 99% agreement respectively, measured using Cohen’s kappa test. The weighted kappa coefficient (κ) was calculated using the Fleiss–Cohen method and reported with 95% confidence intervals (CIs). Reliability was categorized as Excellent (κ > 0.75), Fair-Good (0.4 ≤ κ ≤ 0.75), or Poor (κ < 0.4). Only assessors with “excellent” reliability participated.

#### Standardization

All procedures were conducted in standardized conditions using the same dental chair and identical equipment to ensure consistent air pressure, temperature and procedural uniformity. One clinician performed all procedures, reducing variability in the handling and manipulation of materials [15]. Treatment protocols strictly followed manufacturer guidelines for material application. The outcome assessors were blinded and adhered to standardized pre-established protocols for DH evaluation [22]. Pain assessment was conducted using a validated Visual Analog Scale (VAS) while D.T. occlusion was quantitatively assessed using scanning electron microscopy (SEM) under predefined, standardized imaging conditions. These measures were meticulously applied to maintain uniformity and reliability in clinical evaluations throughout the study.

#### Preparation phase

##### Prophylaxis

Pre-treatment prophylaxis using a fluoride-free paste (Pressage, SHOFU, Japan) applied with a polishing nylon brush (Microdont, São Paulo, SP, Brazil) attached to a low-speed handpiece (Contra T4 HP, Sirona, Germany). Surfaces were thoroughly rinsed with air-water spray and gently dried with cotton to remove debris and moisture, ensuring an optimal surface preparation.

##### Field isolation

Field isolation was achieved using a lip and cheek retractor *(*OptiView Lip and Cheek Retractor, Kerr, Switzerland), cotton rolls, and a saliva ejector. Teeth Surfaces were carefully dried using sterile gauze instead of air blast to prevent triggering hypersensitivity. This isolation method ensured maintaining a dry, controlled environment for material application.

### Treatment phase

#### Material application

PRG Barrier Coat: Mixed one drop active solution with base within 2-minute working time was applied in thin layer cervical-to-incisal, left undisturbed for a minimum of 3 s, light-cured for 10 s using a dental curing unit with an output of ≥ 1000 mW/cm² and an irradiation wavelength of 440–490 nm in standard mode. After curing, the uncured layer was gently removed by rubbing the coated surface with a moistened cotton ball [17, 22]. If the applied mixture became contaminated, it was removed with gauze, and the application procedure was repeated [6, 13]. Participants were instructed to avoid consuming staining foods/drinks for 3 days.

Embrace varnish: Single-use premixed varnish (0.4 mL) applied in thin film. Participants were instructed to avoid consuming hard foods, hot liquids, or alcohol for 3–4 h, and brushing/flossing for at least 4 h, as these actions could prematurely remove the varnish.

Duraphat varnish: Mixed single-use suspension applied in thin layer via painting/dabbing. Participants were instructed to avoid brushing/flossing, solid foods, and alcohol for for at least four hours for optimal adherence and effectiveness.

**Fig. 2:**
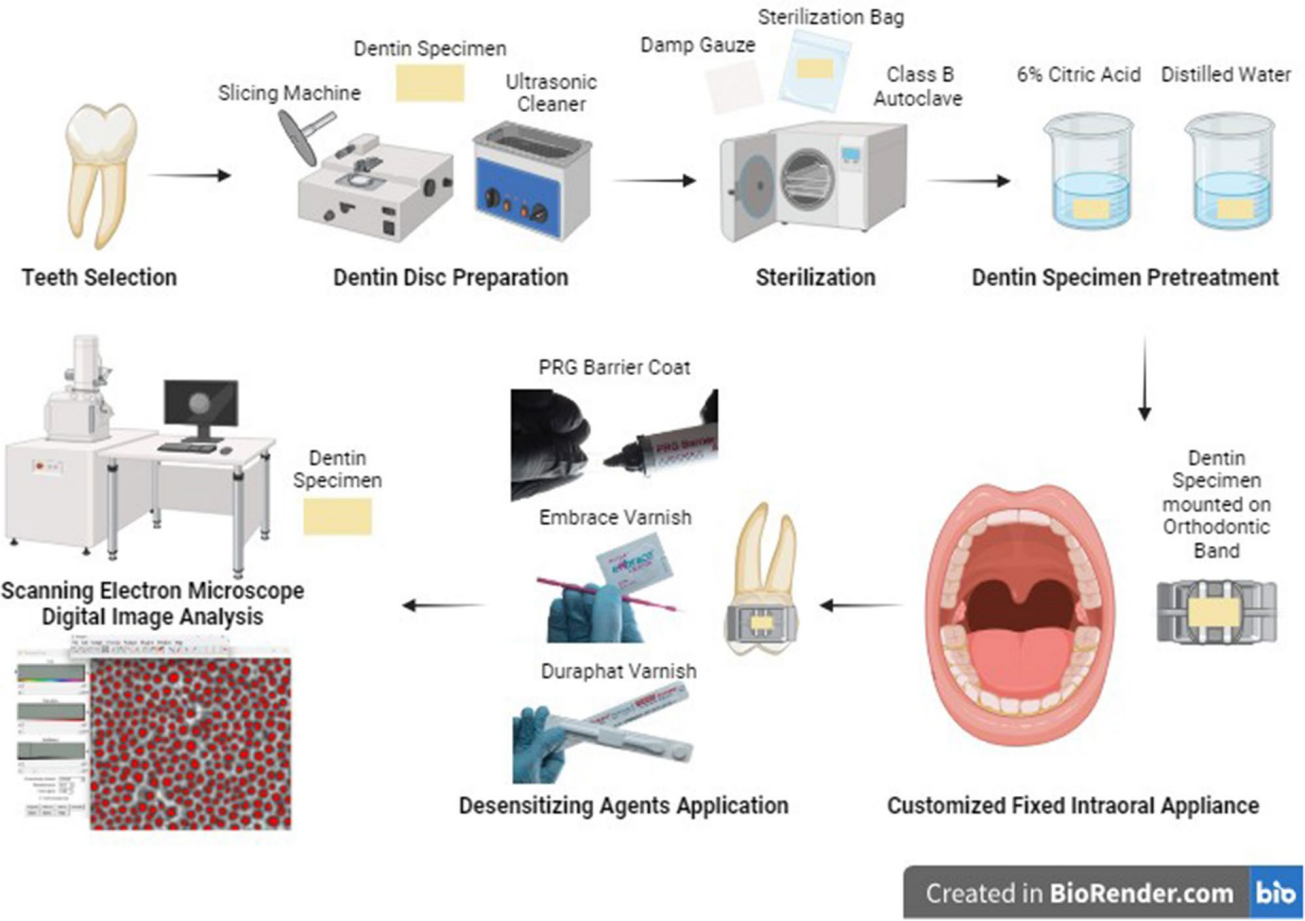
Schematic representation of the in-situ experimental protocol. The diagram illustrates sequential steps of teeth selection, dentin specimen preparation, sterilization, pretreatment, fabrication of customized fixed intraoral appliances, application of desensitizing agents, and subsequent SEM imaging with quantitative image analysis. Created in BioRender. Abd ELFattah, H. (2025) https://BioRender.com/a21a075

In situ **methodology** (Fig. 2).

#### Selection of extracted teeth

A total of 63 extracted sound human molars were collected and stored in 0.2% thymol at 4 °C. All teeth were cleaned of soft and hard deposits using a hand scaler (Dentsply, Germany) and carefully examined under a 6 × magnifying lens (Univet, Italy). To facilitate subsequent preparation procedures, each tooth was mounted in an acrylic block.

#### Preparation of dentin discs

The enamel surfaces of the selected teeth were sectioned both buccolingually and mesiodistally using a diamond disk (MTI, Richmond, CA, USA) mounted on a low-speed microslicing machine (IsoMet™ 4000, Buehler, IL, USA) under continuous water cooling. The occlusal enamel was removed, followed by a second cut 1 mm apical to the exposed dentin, yielding centralized dentin discs with a standardized thickness of 1 mm. Each disc was trimmed to precise dimensions of 6 × 4 × 1 ± 0.05 mm, which were verified using a digital caliper (Silverline Tools, UK). The test surface of each disc was marked with a permanent marker to ensure proper orientation during the experimental procedures [23]. To standardize the smear layer, the dentin discs were polished using 600-grit silicon carbide paper for 20 s, followed by ultrasonication in distilled water at 50 W power for 5 min using an ultrasonic cleaner (FSF 080 S, FAITHFUL Instrument Co., China) [24].

#### Sterilization of dentin specimens

Dentin samples were carefully wrapped in damp gauze to prevent desiccation during processing and individually sealed in sterilization bags (Defend, Shenzhen, China), coded for identification purposes. Sterilization was conducted using a Class B autoclave (Melag, Berlin, Germany) at 121 °C for 20 min to ensure proper asepsis [25].

#### Dentin specimen pretreatment (Stage I - Demineralization Process)

To simulate hypersensitive dentin, each sample was immersed in 6% citric acid (pH 2.0) for two minutes to create open dentinal tubules. Samples were thoroughly rinsed with distilled water (pH 7.0) for one minute to remove residual acid. The samples were air-dried in a sterile Petri dish then returned to their respective sterilization bags [7].

#### Scanning electron microscopy (SEM) analysis

A rectangular area at the center of each dentin sample was marked as the region of interest for scanning electron microscopy (SEM) analysis to ensure standardization. Each sample served as its own control, enabling direct comparison of surface changes before and after the application of desensitizing agents. High-resolution images were captured at 2000× magnification using an environmental SEM (Quanta 3D 200i, FEI, USA). Digital image analysis was performed using ImageJ software (National Institute of Health, Bethesda, USA) to facilitate both qualitative and quantitative evaluations of dentinal tubule surface area and occlusion patterns.

#### Fabrication of customized intraoral appliance

Orthodontic separators (Ortho Technology, Florida, USA) were positioned mesially and distally to the upper first molar created temporary spacing. Alginate impressions (Zhermack, Italy) were taken, and study casts were prepared using hard dental stone (Hinrichs, Germany).

Customized intraoral appliance was fabricated by welding a modified orthodontic band sheet (Dentaurum, Germany) to standard orthodontic band using a laser welder (Sirolaser, Dentsply, Germany). The dentin sample was securely fixed within the appliance using a 0.010-inch stainless steel ligature wire (Ormco, Mexico). The appliance was thoroughly inspected for any surface irregularities or interference. Necessary corrections were made, including polishing, to ensure proper fit and comfort for the participant.

#### Intraoral cementation of customized intraoral appliance

The Customized appliance was cemented using a fluoride-free restorative material (TG, London, UK), exposing only the working dentin surface to the oral environment. Follow-up visits were conducted to ensure adherence, monitor for any adverse effects, and address participant concerns or complaints.

#### Application of desensitizing material (Stage II - Treatment Phase)

Dentin samples were gently dried with absorbent paper simulating clinical conditions [5], followed by manufacturer-specified material application.

#### Specimen collection and analysis

Following the 8-week treatment period, dentin samples were carefully retrieved from the intraoral appliances. Samples were transferred to labeled Eppendorf tubes corresponding to participant identification numbers to maintain traceability. Scanning electron microscopy (SEM) analysis was performed to evaluate surface characteristics before and after 8-week treatment.

#### Cleaning and Preparation of specimens

Dentin samples were ultrasonically cleaned to remove surface deposits. Samples were mounted on aluminum stubs of standard diameter using carbon double-sided sticky tape to ensure stability during imaging.

#### Qualitative and quantitative analysis of dentinal tubule patency

An environmental scanning electron microscope (Quanta 3D 200i, FEI Company, USA) equipped with an energy dispersive X-ray analysis (EDX) unit (Energy Dispersive X-ray Analyses/Thermofisher Pathfinder) was used to analyze the samples. Operating parameters were set at 30 kV accelerating voltage, Gun.1 nm resolution, and 2000× magnification. Surface morphology and dentinal tubule patency were evaluated qualitatively through SEM photomicrographs. Quantitative analysis utilized ImageJ software (National Institute of Health, Bethesda, USA) to measure patent dentinal tubule surface area at both pre-treatment (Stage I) and post-treatment (Stage II after 8-week) stages, with three SEM images analyzed per sample.

#### Outcome measures

##### Primary outcome

Pain intensity was measured using VAS for tactile, evaporative, and thermal stimuli at baseline, 3 min, 2 weeks, 4 weeks, and 8 weeks.

##### Secondary outcome

Patent dentinal tubule surface area was evaluated pre- treatment and post-treatment (after 8-week), with percentage change calculated to quantify treatment effectiveness in dentinal tubules occlusion.

### Follow-up procedures

Standardized dentin hypersensitivity assessments were conducted at predetermined intervals under consistent conditions.

Regular follow-up visits were conducted to monitor gingival health, ensure oral hygiene adherence, ensure compliance with dietary recommendations, and prevent dropouts. These measures improved participant retention, promoted consistent adherence to study protocols, and enhanced the reliability of treatment outcome assessments [7].

### Statistical analysis

Data normality was assessed using Shapiro-Wilk test. Continuous data are presented as mean, standard deviation (SD), median, minimum (min), and maximum (max) values. For comparisons of nonnormally distributed data between multiple nonrelated samples, Kruskal–Wallis test was employed. For comparisons within multiple related samples, Friedman test was used, followed by Dunn’s post hoc test for pairwise analyses. Spearman’s correlation coefficient was calculated to evaluate relationships between nonparametric continuous variables. Categorical data are presented as frequencies (N) and percentages (%) and were analyzed using chi-square test. The significance level for all statistical tests was set as 0.05.

A post-hoc power analysis was conducted to assess the adequacy of the sample size and the strength of the observed effect. Based on the findings of pain intensity following tactile stimulation after 2 months of the current study, the partial Eta squared effect size (ƞ²) was 0.865, indicating a large effect size. The actual power was found to be 1.0, confirming that the study had sufficient statistical power to detect significant differences among the treatment groups.

All statistical analyses were performed using SPSS software (IBM SPSS Statistics for Windows, Version 25.0, IBM, Armonk, NY, USA).

## Results

### Demographic data

Demographics showed comparable distribution across groups. Mean ages [± SD] were 36.4 [± 4.8] (PRG), 35.8 [± 5.8] (Embrace), and 36.7 [± 5.5 years] (Duraphat) (*p* = 0.853). Gender distribution (*p* = 0.639) and tooth type distribution between anterior and premolar teeth (*p* = 0.195) were balanced across groups, establishing homogeneous baseline characteristics (Tables 3 and 4).

**Table 3:** Age distribution and comparison between treatment groups

Descriptive statistics	PRG	Embrace	Duraphat	*p*-value
Mean [± SD] (years)	36.4 [± 4.8]	35.8 [± 5.8]	36.7 [± 5.5]	0.853
Median (Range) (years)	37 [28–44]	37 [26–45]	38 [27–46]	

* Statistically Significant at *p* = 0.05

**Values are presented as mean [± SD] and median (range). Group comparisons were conducted using Kruskal-Wallis test to assess differences in age distribution among the treatment groups

**Table 4:** Gender and tooth distribution across treatment groups

		PRG		Embrace		Duraphat		*p*-value
		**N**	**%**	**N**	**%**	**N**	**%**	
Gender	Males	11	52.4%	8	38.1%	8	47.1%	0.639
	Females	10	47.6%	13	61.9%	9	52.9%	
Teeth	Anterior teeth	16	76.2%	12	57.1%	12	57.1%	0.195
	Premolar teeth	5	23.8%	9	42.9%	9	42.9%	

* Statistically Significant at *p* = 0.05

** Values are presented as frequency (percentage). Group comparisons were conducted using Chi-square test to assess differences in gender and tooth distribution among treatment groups

### Pain intensity

#### Tactile test

Baseline pain intensity showed no significant differences between groups (*p* = 0.820). PRG demonstrated significantly lower pain scores from 3 min to 8 weeks compared to other treatments (*p* < 0.001). All groups showed significant intragroup pain reduction from baseline (*p* < 0.001) (Table 5), with PRG achieving highest reduction (94.9 [± 6.1] %), followed by Embrace (64.3 [± 8.1] %) and Duraphat (45.4 [± 6.6] %) (*p* < 0.001) (Table 6).

**Table 5:** Pain intensity scores following tactile testing at different time intervals between and within treatment groups

		PRG	Embrace	Duraphat	*p*-value
Baseline	Mean [± SD]	8.7^aA^ [± 1.0]	8.4^aA^ [± 1.0]	8.5^aA^ [± 0.7]	0.820
	Median (Range)	8 (7–10)	8 (6–10)	8 (7–10)	
3 min	Mean [± SD]	0^aB^ [± 0.2]	5.7^bB^ [± 0.9]	6.1^bB^ [± 0.7]	< 0.001*
	Median (Range)	0 (0–1)	5 (5–8)	6 (5–8)	
2 weeks	Mean [± SD]	0.1^aB^ [± 0.4]	1.5^bC^ [± 0.7]	3.2^cC^ [± 0.7]	< 0.001*
	Median (Range)	0 (0–1)	1 (0–3)	3 (2–5)	
4 weeks	Mean [± SD]	0.2^aB^ [± 0.4]	2.3^bCD^ [± 0.7]	3.5^cC^ [± 0.7]	< 0.001*
	Median (Range)	0 (0–1)	2 (1–4)	3 (2–5)	
8 weeks	Mean [± SD]	0.4^aB^ [± 0.5]	3.0^bD^ [± 0.8]	4.7^cD^ [± 0.8]	< 0.001*
	Median (Range)	0 (0–1)	3 (2–5)	4 (4–6)	
	*p-*value	< 0.001*	< 0.001*	< 0.001*	

* Statistically Significant at *p* = 0.05

** Values are presented as mean [± SD] and median (range). Statistical significance for between-group comparisons (same row) was determined using Kruskal-Wallis test followed by Dunn’s post hoc test for pairwise comparisons. Within-group comparisons (same column) were analyzed using Friedman test followed by Dunn’s post hoc test for pairwise comparisons

*** Different lowercase letters (a, b, c) indicate significant differences between groups at each time point

**** Different uppercase letters (A, B, C, D) denote significant differences within groups over time

**Table 6:** Pain intensity scores following evaporative testing at different time intervals between and within treatment groups

		PRG	Embrace	Duraphat	*p*-value
Baseline	Mean [± SD]	8.8^aA^ [± 1.0]	8.9^aA^ [± 1.1]	8.9^aA^ [± 0.9]	0.946
	Median (Range)	9 (7–10)	9 (6–10)	9 (7–10)	
3 min	Mean [± SD]	0.1^aB^ [± 0.3]	6.1^bB^ [± 0.8]	6.6^bB^ [± 0.7]	< 0.001*
	Median (Range)	0 (0–1)	6 (5–8)	7 (6–8)	
2 weeks	Mean [± SD]	0.2^aB^ [± 0.4]	2.3^bC^ [± 0.9]	3.5^cC^ [± 0.7]	< 0.001*
	Median (Range)	0 (0–1)	2 (0–4)	3 (2–5)	
4 weeks	Mean [± SD]	0.5^aB^ [± 0.5]	2.5^bCD^ [± 0.8]	4.3^cC^ [± 0.6]	< 0.001*
	Median (Range)	1 (0–1)	2 (1–4)	4 (3–6)	
8 weeks	Mean [± SD]	0.5^aB^ [± 0.5]	3.3^bD^ [± 0.7]	5.2^cD^ [± 0.7]	< 0.001*
	Median (Range)	1 (0–1)	3 (2–5)	5 (4–6)	
	*p-*value	< 0.001*	< 0.001*	< 0.001*	

* Statistically Significant at *p* = 0.05

** Values are presented as mean [± SD] and median (range). Statistical significance for between-group comparisons (same row) was determined using Kruskal-Wallis test followed by Dunn’s post hoc test for pairwise comparisons. Within-group comparisons (same column) were analyzed using Friedman test followed by Dunn’s post hoc test for pairwise comparisons

#### Evaporative test

Pain scores were comparable at baseline (*p* = 0.946). PRG showed significantly lower pain intensity from 3 min onward versus other treatments (*p* < 0.001). All groups demonstrated significant intragroup pain reduction over time (*p* < 0.001) (Table 7), with PRG achieving highest reduction (94.0 [± 6.0] %), followed by Embrace (62.0 [± 7.0] %) and Duraphat (41.4 [± 6.6] % (*p* < 0.001) (Table 6).

**Table 7:** Pain intensity scores following thermal testing at different time intervals between and within treatment groups

		PRG	Embrace	Duraphat	*p*-value
Baseline	Mean [± SD]	8.8^aA^ [± 1.0]	9.0^aA^ [± 1.0]	9.0^aA^ [± 0.8]	0.837
	Median (Range)	9 (7–10)	9 (7–10)	9 (8–10)	
3 min	Mean [± SD]	0.1^aB^ [± 0.4]	6.3^bB^ [± 0.8]	6.7^bB^ [± 0.8]	< 0.001*
	Median (Range)	0 (0–1)	6 (5–8)	7 (6–9)	
2 weeks	Mean [± SD]	0.3^aB^ [± 0.5]	2.4^bC^ [± 0.8]	3.6^cC^ [± 0.7]	< 0.001*
	Median (Range)	0 (0–1)	2 (1–4)	3 (3–5)	
4 weeks	Mean [± SD]	0.6^aB^ [± 0.5]	2.6^bC^ [± 0.9]	4.4^cCD^ [± 0.7]	< 0.001*
	Median (Range)	1 (0–1)	2 (1–5)	4 (3–6)	
8 weeks	Mean [± SD]	0.6^aB^ [± 0.5]	3.5^bD^ [± 0.6]	5.4^cD^ [± 0.7]	< 0.001*
	Median (Range)	1 (0–1)	3 (3–5)	5 (4–6)	
	*p-*value	< 0.001*	< 0.001*	< 0.001*	

* Statistically Significant at *p* = 0.05

** Values are presented as mean [± SD] and median (range). Statistical significance for between-group comparisons (same row) was determined using Kruskal-Wallis test followed by Dunn’s post hoc test for pairwise comparisons. Within-group comparisons (same column) were analyzed using Friedman test followed by Dunn’s post hoc test for pairwise comparisons

*** Different lowercase letters (a, b, c) indicate significant differences between groups at each time point

**** Different uppercase letters (A, B, C, D) indicate significant differences within groups over time

*** Different lowercase letters (a,* b*,* c) indicate significant differences between groups at each time point.*

**** Different uppercase letters (A, B, C, D) denote significant differences within groups over time.

#### Thermal test

Pain intensity was comparable across groups at baseline (*p* = 0.837). PRG demonstrated significantly lower pain scores from 3 min to 8 weeks compared to other treatments (*p* < 0.001). All groups showed significant intragroup pain reduction over time (*p* < 0.001) (Table 8), with PRG exhibiting highest reduction (93.5 [± 5.9] %), followed by Embrace (60.4 [± 6.4] %) and Duraphat (40.3 [± 5.0] % (*p* < 0.001) (Table 6).

**Table 8:** Comparison of pain reduction percentages between treatment groups

	Descriptive statistics	PRG	Embrace	Duraphat	*p*-value
Tactile	Mean [± SD] (%)	94.9^a^ [± 6.1]	64.3^b^ [± 8.1]	45.4^c^ [± 6.6]	< 0.001*
	Median (Range) (%)	100 (85.7–100)	62.5 (50–77.8)	44.4 (33. 3–55.6)	
Evaporative	Mean [± SD] (%)	94.0^a^ [± 6.0]	62.0^b^ [± 7.0]	41.4^c^ [± 6.6]	< 0.001*
	Median (Range) (%)	90 (85.7–100)	62.5 (50–80)	40 (28.06–55.06)	
Thermal	Mean [± SD] (%)	93.5^a^ [± 5.9]	60.4^b^ [± 6.4]	40.3^c^ [± 5.0]	< 0.001*
	Median (Range) (%)	90 (85.7–100)	60 (50–70)	40 (33.3–50)	

* Statistically Significant at *p* = 0.05

** Values are presented as mean [± SD] and median (range) percentages. Statistical significance for between-group comparisons (same row) was determined using Kruskal-Wallis test for pain reduction percentage between groups

*** Different lowercase letters (a, b, c) indicate significant differences between groups

### Dentinal tubule patency/occlusion and SEM analysis

#### Quantitative analysis

Quantitative SEM analysis revealed a significant reduction in patent dentinal tubule surface area following treatment. The PRG group demonstrated the most substantial change, with the percentage of patent tubule area decreasing from (21.0 [± 2.7] %) at baseline to (0.6 [± 0.5] %) after 8 weeks. Embrace™ showed moderate occlusion (26.2 [± 4.5] %) to (8.0 [± 2.5] %) while Duraphat exhibited the least reduction from (23.2 [± 5.4] %) to (11.9 [± 3.1] %) (Table 9). The percentage of change was significantly higher in the PRG group (96.9 [± 2.5] %) compared to Embrace™ (69.7 [± 5.7] %) and Duraphat (48.3 [± 7.6] %) (*p* < 0.001) (Table 10).

**Table 9:** Comparison of patent dentinal tubule surface area percentages between and within treatment groups after stage I and stage II

	Descriptive statistics	PRG	Embrace	Duraphat	*p*-value
Stage I	Mean [± SD] (%)	21.0^aA^ [± 2.7]	26.2^bA^ (± 4.5)	23.2^abA^ [± 5.4]	0.001*
	Median (Range) (%)	20.9 (16.8–25.3)	24.7 (20.1–36.2)	25.1 (14.2–29.9)	
Stage II	Mean [± SD] (%)	0.6^aB^ [± 0.5]	8.0^bB^ [± 2.5]	11.9^cB^ [± 3.1]	< 0.001*
	Median (Range) (%)	0.5 (0.0–1.5)	6.5 (5.3–13.0)	11.3 (6.9–17.2)	
	*p-*value	< 0.001*	< 0.001*	< 0.001*	

* Statistically Significant at *p* = 0.05

**Values are presented as mean [± SD] and median (range) percentages. Statistical significance for between-group comparisons (same row) was determined using Kruskal-Wallis test followed by Dunn’s post hoc test for pairwise comparisons. Within-group comparisons (same column) were analyzed using Friedman test followed by Dunn’s post hoc test for pairwise comparisons

***Different lowercase letters (a, b, c) denote significant differences between groups at each time point

****Different uppercase letters (A, B, C, D) denote significant differences within groups over time

**Table 10:** Comparison of percentage of change in patent dentinal tubule surface area between treatment groups

Descriptive statistics	PRG	Embrace	Duraphat	*p*-value
Mean [± SD] (%)	96.9^a^ [± 2.5]	69.7^b^ [± 5.7]	48.3^c^ [± 7.6]	< 0.001*
Median (Range) (%)	97.7 (93.4–100)	70.0 (59.5–79.7)	47.5 (31.6–59.8)	

* Statistically Significant at *p* = 0.05

**Values are presented as mean [± SD] and median (range) percentages. Statistical significance for between-group comparisons (same row) was determined using Kruskal-Wallis test for percentage of change in patent dentinal tubule surface area

***Different lowercase letters (a, b, c) denote significant differences between groups

### Qualitative analysis

**Fig. 3:**
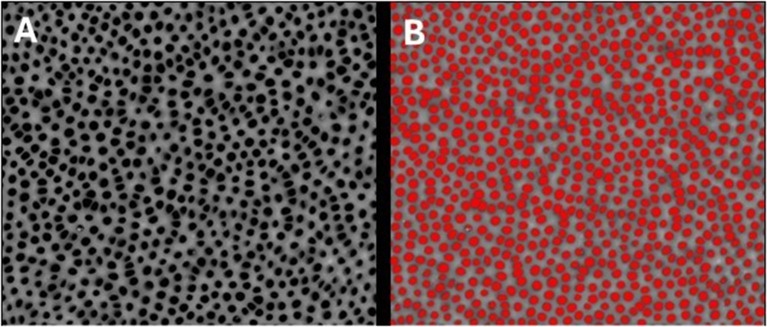
SEM images of demineralized dentin following Stage I treatment. **(A)** SEM photomicrograph of demineralized dentin after Stage I, showing exposed dentinal tubules following tremoval of smear layer. **(B)** Analyzed SEM photomicrograph of demineralized dentin after Stage I, illustrating quantification of patent dentinal tubule surface area using image analysis software. Scale bar = 50 μm

**Fig. 4:**
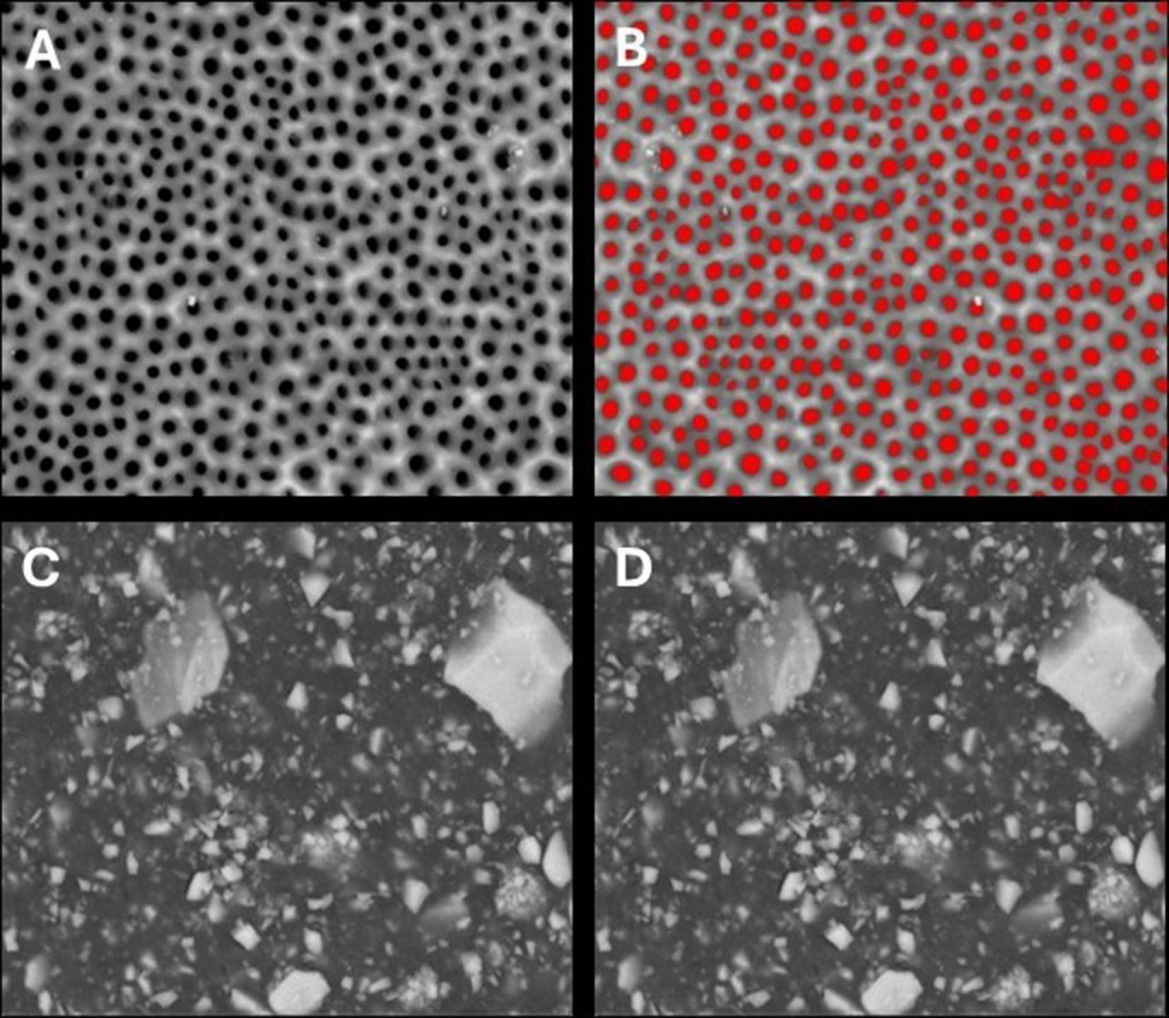
SEM images of dentinal tubules before and after treatment with PRG barrier coat. **(A)** SEM image of demineralized dentin after Stage I, showing exposed dentinal tubules following removal of smear layer. **(B)** Analyzed SEM image of demineralized dentin after Stage I, showing quantified patent dentinal tubule surface area using image analysis software. **(C)** SEM image of demineralized dentin after Stage II, treated with PRG Barrier Coat, showing dentinal tubule occlusion. **(D)** Analyzed SEM image of PRG-treated dentin after Stage II, illustrating tubule occlusion quantification. Scale bar = 50 μm

**Fig. 5:**
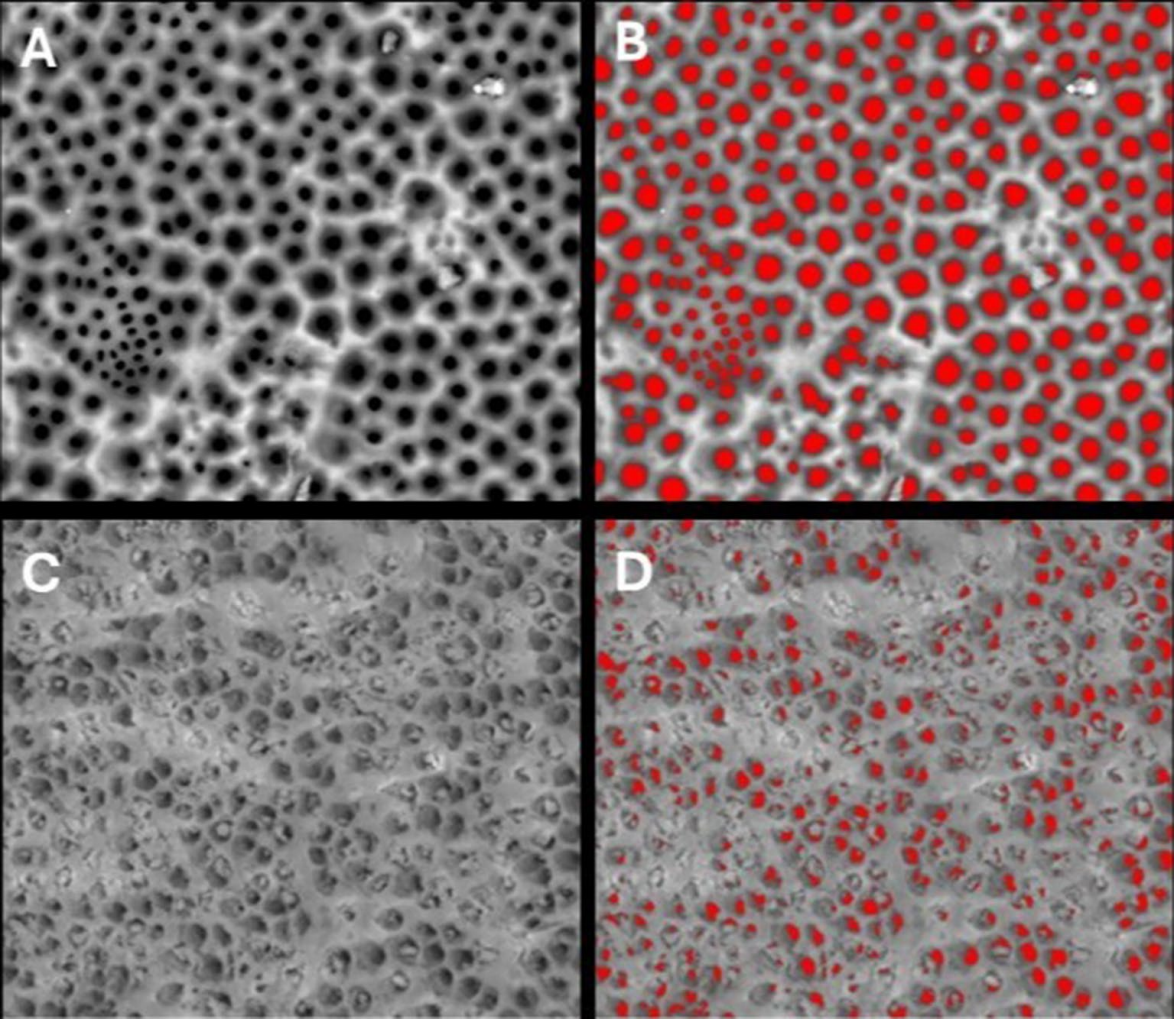
SEM images of dentinal tubules before and after treatment with Embrace varnish. **(A)** SEM image of demineralized dentin after Stage I, showing exposed dentinal tubules following removal of smear layer. **(B)** Analyzed SEM image of demineralized dentin after Stage I, showing quantified patent dentinal tubule surface area using image analysis software. **(C)** SEM image of demineralized dentin after Stage II, treated with Embrace varnish, showing dentinal tubule occlusion. **(D)** Analyzed SEM image of Embrace-treated dentin after Stage II, illustrating tubule occlusion quantification. Scale bar = 50 μm

**Fig. 6:**
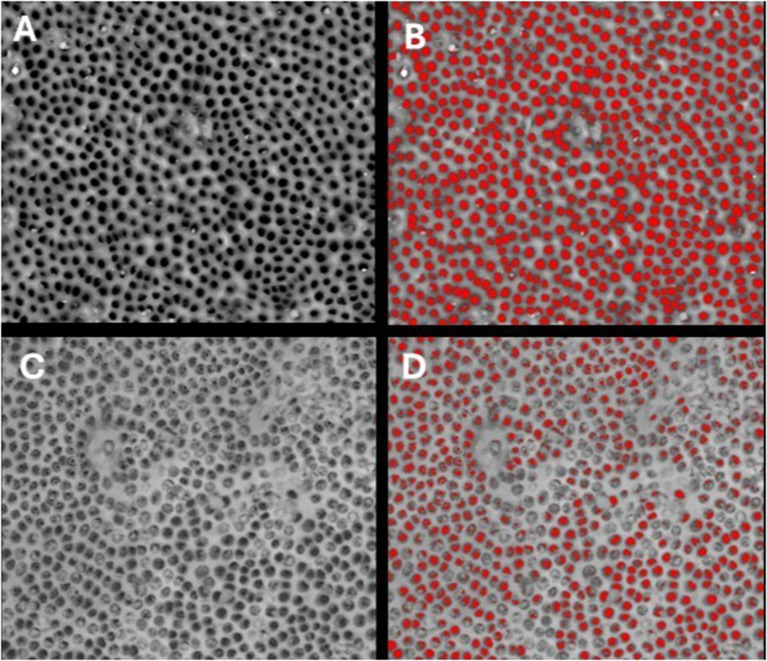
SEM images of dentinal tubules before and after treatment with Duraphat varnish. **(A)** SEM image of demineralized dentin after Stage I, showing exposed dentinal tubules following tremoval of smear layer. **(B)** Analyzed SEM image of demineralized dentin after Stage I, showing quantified patent dentinal tubule surface area using image analysis software. **(C)** SEM image of demineralized dentin after Stage II, treated with Duraphat varnish, showing dentinal tubule occlusion. **(D)** Analyzed SEM image of Duraphat-treated dentin after Stage II, illustrating tubule occlusion quantification. Scale bar = 50 μm

SEM analysis revealed distinct occlusion patterns across treatments. Pretreated samples showed patent dentinal tubules without smear layer (Fig. 3). PRG Barrier Coat demonstrated complete occlusion with dense, angular deposits indicating bioactive remineralization (Fig. 4). Embrace Varnish exhibited partial to substantial occlusion with varying coverage, showing fully and partially occluded tubules from xylitol-coated calcium phosphate deposition (Fig. 5). Duraphat displayed minimal occlusion with sparse deposits around tubule openings, suggesting limited durability (Fig. 6).

### Correlation between pain intensity reduction percentage and percentage of change in patent dentinal tubules surface area

**Fig. 7:**
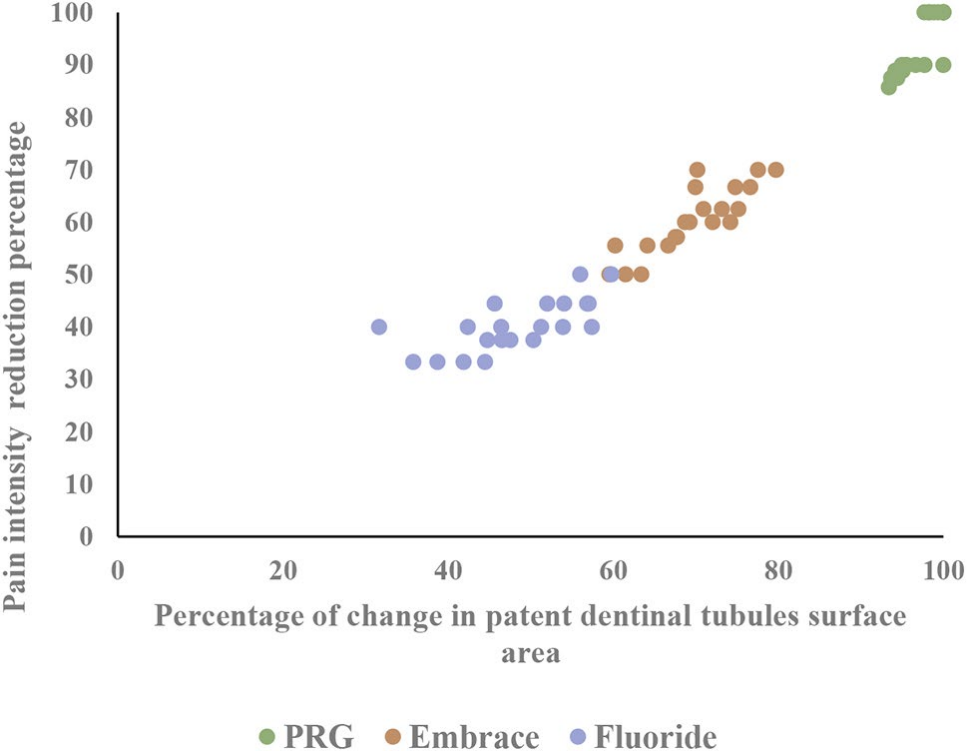
Scatter plot illustrating correlation between pain intensity reduction percentage and percentage of change in patent dentinal tubule surface area across all treatment groups (PRG Barrier Coat, Embrace varnish, and Duraphat varnish)

Spearman rank-order correlation was used to examine the relationship between pain intensity reduction percentage and percentage of change in patent dentinal tubules surface area. A strong positive correlation was observed between pain intensity reduction percentage and percentage of change in patent dentinal tubules surface area across all treatment groups (*r*_*s*_ = 0.98, *n* = 21, *p* < 0.001). This correlation was most pronounced in the PRG group (rs = 0.88, *p* < 0.001), followed by Embrace (*rs* = 0.879, *p* < 0.001) and Duraphat (*rs* = 0.713, *p* < 0.001) (Fig. 7).

## Discussion

This study revealed distinct patterns of effectiveness among desensitizing agents through both comprehensive surface analysis and clinical evaluation. The observed differences in treatment outcomes between PRG Barrier Coat and fluoride varnishes can be attributed to their distinct compositions and mechanisms of action. Fluoride varnishes primarily function by forming transient calcium fluoride-like deposits on the dentin surface, which gradually release fluoride ions to promote remineralization and tubule occlusion [19]. However, these deposits are susceptible to dissolution and mechanical removal over time, necessitating repeated applications for sustained efficacy [9, 15]. In contrast, PRG Barrier Coat, as a light-cured resin-based material, adheres to dentin via chemical interaction and mechanical retention, forming a durable, bioactive layer that resists acid and mechanical challenges [5]. This structural integrity allows for sustained ion release and prolonged tubule occlusion, contributing to its superior clinical performance [26–28]. While the differences in outcomes between these two materials may be anticipated based on their inherent properties, direct comparative evaluation is essential for determining their relative effectiveness in real-world conditions and guiding clinical decision-making in dentin hypersensitivity management.

PRG Barrier Coat demonstrated superior dentin hypersensitivity relief, which may be attributed to its bioactive S-PRG fillers. These fillers facilitate sustained ion release, supporting dentinal tubule occlusion and potential remineralization effects [5]. Previous studies have suggested that S-PRG fillers contribute to the formation of fluorapatite and strontium apatite, contributing to dentin remineralization and structural integrity [5, 26, 27].

SEM analysis confirmed the formation of a robust bioactive layer characterized by nearly complete tubule occlusion, effectively resisting both acid and mechanical challenges [5, 28–30]. These observations align with previous studies demonstrating the efficacy of S-PRG fillers in maintaining dentinal tubule occlusion under erosive-abrasive conditions [24, 28–30]. However, these findings contrast with Mosquim et al. (2022), who reported limited dentin permeability reduction with S-PRG varnish compared to sodium fluoride varnish [31], highlighting the limitations of in vitro models in replicating clinical conditions, particularly regarding the protective role of saliva and long-term bioactive effects.

The sustained efficacy of PRG in providing prolonged pain relief corresponds with broader literature emphasizing its bioactive properties [13, 17, 26]. However, variations in onset timing have been noted across studies, potentially influenced by differences in study populations and baseline sensitivity levels [13, 26]. Additionally, some investigations have suggested a potential three-month relapse, which could be attributed to environmental factors including saliva exposure, brushing habits, and variability in pain assessment methods [6].

Embrace varnish exhibited moderate efficacy through its xylitol-coated calcium phosphate formulation, facilitating gradual crystal deposition and enhanced remineralization. Xylitol’s role in enhancing calcium and phosphate ion bioavailability has been highlighted in prior studies, supporting its ability to promote acid-resistant fluoroapatite formation [32].

While certain investigations ranked it second in remineralization depth [33] and reported superior initial fluoride uptake with higher calcium-to-phosphate ratio [34], Limitations related to sustained fluoride release and environmental resistance suggest a need for frequent reapplications [9, 35]. Reports on delayed onset of action vary, contrasting with our findings of better initial pain relief, potentially reflecting differences in application protocols and participant adherence [21].

The unique resin-based composition of Embrace varnish promotes localized ion supersaturation, which contributes to its remineralizing properties [36].

These findings align with comparative studies demonstrating superior tubule occlusion compared to conventional fluoride varnishes reinforcing its effectiveness in DH management [37, 38]. The synergistic combination of calcium phosphate and fluoride facilitates acid-resistant fluoroapatite formation [39]. While enhanced by xylitol’s remineralization properties, as observed by Cardoso et al. (2016) and Gargouri et al. (2018) [40, 41], limitations in its long-term performance is influenced by its fluoride release profile, necessitating further evaluation of sustained effects [42].

Duraphat varnish demonstrated the least effective pain relief, with noticeable efficacy decline beyond 4 weeks, attributed to its reliance on transient calcium fluoride deposits. This aligns with findings that highlight the short-term efficacy of fluoride-based desensitizers [15, 43]. SEM imaging confirmed minimal tubule occlusion, consistent with studies reporting deposit detachment under oral conditions [44, 45]. The superficial fluoride deposits exhibited vulnerability to brushing and acidic environments, limiting long-term efficacy.

A robust positive correlation was observed between dentinal tubule occlusion percentage and pain reduction across all groups, reinforcing the hydrodynamic theory of dentin hypersensitivity [46]. PRG Barrier Coat’s superior correlation highlighted its consistent and durable tubule occlusion capacity, establishing it as the most effective treatment in this study.

The null hypothesis (H₀), which proposed that there would be no statistically significant differences in pain intensity (primary outcome) among PRG Barrier Coat, Embrace varnish, and Duraphat varnish at at 3 min, 2-week, 4-week, and 8-week intervals, was rejected. The findings demonstrated statistically significant differences among the three treatment groups, with PRG Barrier Coat exhibiting the most substantial reduction in pain intensity and dentinal tubule occlusion, followed by Embrace varnish, while Duraphat varnish exhibited the least effectiveness (*p* = 0.05). These results confirm that the tested materials do not exhibit equivalent performance, thereby rejecting the null hypothesis.

This study has several strengths that enhance its scientific validity and clinical relevance in dentin hypersensitivity management. The comprehensive evaluation of both subjective and objective outcome measures, integrating pain intensity reduction (VAS) and dentinal tubule occlusion (SEM analysis) ensures a thorough assessment of treatment effectiveness. The integration of in vivo and in situ methodologies, combining the advantages of clinical pain assessment with controlled laboratory conditions. The in vivo component enabled real-time evaluation of pain reduction in patients, ensuring clinical applicability, while the in situ model facilitated direct SEM visualization of dentinal tubule occlusion. This hybrid approach strengthens the study by minimizing inter-subject variability while preserving real-world relevance. The in situ model further enabled direct imaging of treated dentin surfaces before and after intervention, providing insights into mechanism of action of desensitizing agents.

The use of a fixed intraoral appliance further strengthens the study as it ensured continuous exposure of dentin specimens to oral environment, thereby closely mimics clinical conditions while allowing for controlled laboratory analysis. Unlike removable appliances, which are subject to inconsistent exposure times and patient compliance issues, the fixed appliance ensured uninterrupted exposure to salivary components, dietary factors, and mechanical brushing forces throughout the study period. Clinically, this study provides quantitative evidence comparing PRG barrier coat with conventional fluoride varnishes, contributing to evidence-based treatment selection for dentin hypersensitivity. The findings support the potential of bioactive resin-based coatings in offering sustained pain relief and tubule occlusion, compared to fluoride-based treatments that require more frequent reapplications.

## Limitations

This study’s limitations include the in-situ model’s inability to fully replicate the complexity of clinical conditions including constant intra-pulpal pressure and dynamic interactions with dentinal fluids. While SEM analysis provided valuable structural insights, incorporating complementary methodologies, such as confocal laser microscopy and elemental analysis (EDEX) could offer a more comprehensive understanding of varnishes’ structural and biochemical effects. Additionally, the single-application protocol may underestimate cumulative benefits of repeated applications, which warrants further exploration. Moreover, the variability in patient oral hygiene compliance, dietary habits and environmental conditions may have influenced varnish performance outcomes though efforts were made to standardize procedures.

### Future directions and recommendations

- Extended trials beyond 8 weeks are needed to assess durability and cumulative treatment effects.
- Molecular investigations into PRG technology’s ion release mechanisms and tubule sealing properties.
- Optimization of fluoride varnish formulations to enhance adhesion, durability, and sustained ion release.
- Exploration of synergistic effects with laser therapy or adjunctive desensitizing treatments.
- Advanced imaging techniques to provide deeper structural insights into treatment mechanisms.

## Conclusion

This study demonstrated that PRG Barrier Coat and Embrace varnish effectively reduced pain intensity and promoted dentinal tubule occlusion, with PRG Barrier Coat exhibiting the most sustained effects. These findings emphasize the clinical significance of dentinal tubule occlusion in management of DH and highlight the potential advantages of bioactive materials over conventional fluoride varnishes. Treatment selection should consider both immediate pain relief and durability of therapeutic effects. Further research is necessary to assess extended clinical outcomes and optimize reapplication protocols for enhanced treatment efficacy.

### Clinical implications

1. PRG Barrier Coat demonstrated sustained pain reduction and enhanced dentinal tubule occlusion, making it a suitable option for patients requiring prolonged symptom relief.
2. Embrace varnish showed moderate efficacy, suggesting its suitability for mild to moderate DH cases, with potential need for periodic reapplications to maintain effectiveness.
3. Duraphat varnish, while providing pain relief and fluoride-mediated protection, exhibited less pronounced tubule occlusion, indicating its potential role as an adjunctive treatment or for short-term DH relief.

## Data Availability

The data supporting the findings of this study are available from the corresponding author, upon reasonable request.
